# Breaking cellular boundaries: molecular mechanisms of tunneling nanotube formation and fusion

**DOI:** 10.1042/BST20250093

**Published:** 2026-03-13

**Authors:** Sevan Belian, Chiara Zurzolo

**Affiliations:** Institut Pasteur, Université Paris Cité, CNRS UMR3691, Unité de Trafic Membranaire et Pathogénèse, 75015 Paris, France

**Keywords:** actin, cell homeostasis, cell-cell communication, membrane fusion, Tunneling nanotubes

## Abstract

Tunneling nanotubes (TNTs) are thin, actin-based membrane bridges that establish direct cytoplasmic continuity between distant cells, enabling the transfer of diverse cargoes ranging from ions and proteins to organelles such as mitochondria. Since their discovery in 2004, TNTs have been identified in numerous cell types and linked to an expanding range of physiological and pathological functions. Yet, their molecular identity and mechanisms of formation remain elusive. The most defining and least understood step in TNT biogenesis is membrane fusion, the process by which TNTs achieve open-ended continuity between cells, and this represents a critical frontier in the field. This review integrates recent advances in TNT biology, emphasizing the interplay between actin cytoskeletal dynamics, plasma membrane composition, and cell adhesion during TNT formation. It also draws mechanistic parallels with established models of membrane fusion, highlighting fundamental principles and shared regulators across fusion systems, many of which have been implicated in TNT functionality. By combining molecular, biophysical, and imaging perspectives, this review proposes a conceptual framework for TNT formation and fusion, identifies major methodological gaps, and outlines future directions to unravel the mechanisms that underlie intercellular cytoplasmic continuity.

## Introduction

Tunneling nanotubes (TNTs) are open-ended, actin-based membrane protrusions that connect cells and allow direct membrane and cytoplasmic continuity. First described in 2004 by Rustom et al. in PC12 cells in 2D culture [1], TNTs have since been shown to mediate the intercellular transfer of a wide array of cargoes, including ions, RNAs, proteins, vesicles, and even entire organelles such as lysosomes and mitochondria (Table 1) [2]. While vesicle and organelle transport are thought to rely on myosin motors, soluble cytosolic components have been shown to diffuse through TNTs, challenging the long-held view of the cell as a discrete, self-contained entity [3–5].

TNTs have been observed across numerous cell types, including immune cells [6,7], epithelial cells [8], neural cells [9,10], cardiac cells [11,12], cancer cells [13], stem cells, mesenchymal cells [14,15], and even between cells of different origins [9,12,16,17] (Table 1).

**Table 1:** Cargoes transported through TNTs in various cell lines

Cargo transferred through TNTs	Cell lines (donor and/or acceptor)	References
Mitochondria	PC12, rat hippocampal neurons and astrocytes, SMCs, MSCs, mouse primary cortical neurons, human neural stem cells, LA-4, 3T3, NHBE, SH-SY5Y, HMC3, primary mesothelioma cells, LP9, A549, Met5A, U87 MG, human primary GBM cells, T-cells, B-cells, RT4, T24, human primary ovarian cancer cells, human primary breast cancer cells, human primary laryngeal cancer cells, Jurkat cells, human corneal epithelial cells, human cancer-associated fibroblasts, human bronchial epithelial cells	[12–14,16,18–44]
Intracellular vesicular compartments (endosomes, autophagosomes, lysosomes, ER-derived vesicles, Golgi-derived vesicles)	CAD, HSPC-derived macrophages, cystinotic fibroblasts, SH-SY5Y, primary human pericytes, ESC-derived astrocytes, primary mouse microglia, primary mouse podocyte, NALM6, SCC-derived cells, Primary human mesothelioma cells, primary retinal neurons, primary photoreceptor neurons, zebrafish embryo, MSC, prostate cancer cells, HEK293T, MDA-MB-231	[1,3,4,13,17,45–62]
Prions (PrPSc)	CAD, mouse primary cerebellar granule neurons, mouse primary embryonic hippocampal neurons, mouse primary bone-marrow derived dendritic cells	[9,60]
α-synuclein	SH-SY5Y, HMC3, Primary human and mouse microglial cells, CAD, HeLa, mouse primary BMVEC, mouse primary pericytes, mouse and human primary astrocyte, U-87 MG, U251, ESC-derived astrocyte	[3,16,17,45,52,57,63–66]
Tau	CAD, mouse and rat primary neurons, SH-SY5Y	[67–69]
Amyloid-β	SH-SY5Y, rat and mouse primary neurons and astrocytes	[43,70,71]
Rhes protein and mHTT polyQ aggregates	Mouse primary neurons, CAD, STHdh	[72–75]
HIV	Monocyte-derived macrophages, primary human macrophages, U87	[76–78]
Influenza	MDCK, A549, Vero, primary human bronchial epithelial cells	[8,79,80]
SARS-CoV-2	Vero E6, SH-SY5Y	[81]
Mycoplasma	NIH3T3	[82]
MicroRNA	K7M2, SKOV3, MC3T3, primary mouse and human SMCs, primary human endothelial cells, T24, RT4	[83–85]
siRNAs	MEFs, HEK293T	[86]
Lipid droplets	HMEC-1, human astrocytes	[87,88]
P-glycoprotein	MCF-7	[89]
EGFP-actin	PC12	[1]
MHC class I	HeLa	[90]
Stress-associated proteins (CLU, YB-1, Hsp27, LC3	PC3, LNCaP	[91]
KRAS	Colon and colorectal cancer cell lines	[92]
H-Ras	B-Cells, H-Cells	[93]
Transferrin receptor	HeLa, NRK, MDA-MB231, SAOS-2	[50]
LST1, RalA	Bladder cancer cells	[94]
Dendra2	Zebrafish embryo	[4]
Nanoparticles	Primary cardiomyocytes, glioblastoma cells, RAW, J774A.1, mouse perivascular macrophages, HeLa, 4T1, A549, HEK293T, SH-SY5Y	[95–100]
Hydrazide probe	SH-SY5Y	[3]
EGFP	Mouse astrocytes and neurons	[101]
Lucifer Yellow (upon GAP junction inhibition)	Intestinal epithelial cells	[5]

In addition to open-ended TNTs, actin-based connections called closed-ended TNTs have also been described. These structures enable electrical coupling via calcium diffusion through gap junctions located at their tip [102,103]. Such closed-ended connections have been implicated in neurovascular coupling, for instance, between pericytes in the mouse retina [104]. While they share structural and functional similarities with open-ended TNTs, they are not able to transfer cargoes larger than the gap junction pore. By contrast, open-ended TNTs can transfer organelles, vesicles, and protein aggregates, a capability that requires the fusion of plasma membranes at their contact sites (Table 1). How such diverse intercellular structures emerge from common cytoskeletal and membrane remodeling pathways remains a major question in cell biology.

Recent *in vivo* studies provide growing evidence for functional nanotube-like connections in the nervous system and other tissues. In the mammalian cortex, dendritic nanotubes (DNTs) have been described between neurons and shown to mediate Ca^2+^ signaling and amyloid-β propagation, although direct visualization of cargo passage through open-ended structures remains unresolved [71]. EGFP transfer from astrocytes to neurons has also been reported to depend on an F-actin-dependent yet non-secretion-based mechanism [101]. Similarly, Myosin-X-dependent tunneling nanotube-like structures were reported to enable mitochondrial transfer from satellite glial cells to sensory neurons in dorsal root ganglia, a process essential for neuronal maintenance and protection against peripheral neuropathy, yet open-ended continuity could not be directly demonstrated *in vivo* [105]. Beyond the nervous system, TNT-like actin-based protrusions were identified in the developing mouse heart, where they physically bridge cardiomyocytes and endocardial cells across the cardiac jelly, enabling the transfer of cytoplasmic proteins and signaling molecules required for Notch-dependent trabecular morphogenesis [106]. In contrast with these mammalian studies, the optical transparency of the zebrafish embryo allowed Korenkova et al. to directly visualize the free diffusion of photoactivated cytosolic proteins and the transfer of mitochondria between connected cells by live microscopy, providing strong evidence for open-ended TNTs *in vivo* [4].

Together, these studies support a conserved role for actin-based nanotubular connections in physiological and pathological intercellular communication, while highlighting persistent technical challenges in resolving membrane continuity in mammalian tissues.

In the following sections, we will review the current knowledge on TNT biogenesis and function, focusing on actin dynamics, cell adhesion, and membrane composition. Special attention will be given to the final and least understood step of TNT formation: tip-to-target membrane fusion.

We will compare these processes with other plasma membrane fusion events in mammals, highlighting key open questions and proposing a hypothetical model of TNT formation and fusion.

## Linking structure to function in TNTs

TNTs lack specific molecular markers. Morphology alone, thin (150–900 nm) actin-based protrusions, is not sufficient to distinguish them from filopodia, from which they might arise, or from retraction fibers. Instead, they are functionally defined by their ability to transfer intracellular material between cells, a property that likely reflects a structural difference: membrane continuity versus closed tip.

Most studies on TNTs are performed in 2D culture systems, where transfer can be more easily observed. In these settings, TNTs appear non-adherent to the substrate and exhibit straight, tensed morphologies, likely due to non-muscle myosin II (NMII)-induced contractility [107], and they can extend over 100 μm, which is much longer than the majority of canonical filopodia, which rarely exceed 5 μm in length. Importantly, there is no evidence that TNTs need to be long to be functional. However, this concept has not been widely considered and is only beginning to emerge [48]. The ease of visualizing cargo movement in longer TNTs may introduce a sampling bias, leading to the underrepresentation of shorter ones. Cells in close proximity tend to display more connections, which are harder to visualize, yet this correlates with increased TNT-mediated transfer [48,54]. It is worth noting that the concept of non-substrate adherence, so relevant to and emphasized in studies on TNTs in 2D cultures, becomes largely irrelevant when applied to 3D environments such as living tissue.

TNT-mediated transfer has been documented for numerous cell types and cargoes (Table 1). Notably, live imaging of patch clamp-injected hydrazide across multiple SH-SY5Y cells via TNTs demonstrated simultaneous cytosolic diffusion across multiple TNT-connected cells [3], while photoactivatable Dendra2 was observed diffusing through CEP55-negative bridges in live zebrafish embryos [4]. In both cases, large proteins diffused rapidly and homogeneously between connected cells, consistent with free diffusion through cytoplasmically continuous TNTs. Similarly, transfer of Lucifer Yellow in intestinal epithelial cells occurred even when gap junctions were inhibited by gap27 or α-glycyrrhetinic acid, suggesting alternative communication pathways, such as open-ended TNTs [5]. Correlative FIB-SEM imaging in human SH-SY5Y neuroblastoma and murine Cath.a-differentiated (CAD) neuronal-like cells confirmed the presence of continuous tunnels open at both ends [10], and correlative TEM observed similar results in HEK293 and MDA-MB-231 breast cancer cells [62]. These finding currently represent the most direct evidence of fusion in TNTs.

Over the past two decades, TNTs have been shown to transfer a broad spectrum of cargo and have been implicated in both physiological and pathological contexts [108]. Yet, their mechanism of formation remains poorly understood, largely due to the lack of specific markers allowing distinguishing them from closed-ended filopodia. This gap in knowledge continues to limit our ability to dissect TNT-specific functions *in vivo* and to evaluate their relevance in disease. In the next section, we examine the current evidence on TNT biogenesis and identify the key experimental challenges that remain.

## Actin regulation of TNT biogenesis

As actin-based structures, TNTs share many features with filopodia [109]. Cryo-EM analyses performed on SH-SY5Y and CAD cells revealed striking similarities: both structures display comparable diameters, hexagonal organization of the actin filaments within the bundles, and interfilament spacing of ∼10 nm [10]. However, what appears as a single TNT in confocal microscopy often consists of a bundle of thinner tubes, termed individual TNTs (iTNTs), averaging ∼123 nm in diameter. While some of these iTNT appear closed-ended, others maintain membrane continuity along their entire length. As non-substrate adherent structures, TNTs possess greater freedom of movement compared with adherent filopodia, which, paired with cadherin-mediated interactions, may explain the frequent formation of such bundles in 2D cultures. Recent work suggests N-cadherin based adhesion could contribute to stability, elongation, or fusion capacity of TNTs [48].

TNTs also differ from filopodia in cargo content. They are often enriched with vesicles and organelles and show fewer F-actin interruptions, suggesting different actin dynamics or interactions with motor proteins [10]. Several factors may underlie these differences, such as the differential enrichments of actin regulators (e.g., tropomyosins, cofilin), or NMII-generated tension in substrate-adherent actin-based protrusions compared with non-adherent ones.

Experimentally, modulating actin regulators can bias cells toward either TNT or filopodia formation. For example, in CAD cells, Arp2/3 inhibition by CK666 reduces branched actin networks, increasing G-actin availability and promoting TNT elongation between cells plated on distant fibronectin-coated micropatterns [54]. Similar results were observed in cells cultured under uniform plating conditions, including human neuronal SH-SY5Y cells [10] and U-87 malignant glioma cells [66]. On the other hand, Arp2/3 inhibition in trabecular meshwork or A549 cells decreased TNT-like connections or TNT-mediated intercellular transfer [55,110]. In SH-SY5Y human neuronal cells and RAW/LR5 macrophages, inhibition of the Arp2/3 upstream regulators N-WASP decreases both TNT-mediated transfer and TNT formation [48,111], suggesting that local and regulated Arp2/3 activity could participate in TNT formation and that cell-type specificity should also be taken into account.

Multiple actin regulators have been implicated in TNT formation (Table 2), often with context-specific effects. These include Rho GTPases (Rac1, Cdc42, RhoA), downstream effectors (ROCK, WASP, WAVE2, PAK1/2), actin nucleators (Arp2/3, formins), and trafficking proteins (Rab11a, Rab8a, Rab35, Rhes), whereas formin agonists such as IMM-01 enhance TNT formation [17,48,49,54,73,75,110–116]. Similarly, the myosin motor Myosin-X has been shown to promote TNT formation in CAD cells, a process dependent on its PH2 domain, which selectively binds to the phosphoinositide PI(3,4,5)P_3_ [117]. Myosin-X is also essential for the intercellular transfer of the HIV-1 accessory protein Nef through TNTs from macrophages to T cells [118].

**Table 2:** Known regulators of TNT formation

Protein name	Main cellular function(s)	Cell type(s) where they were shown to positively regulate TNTs	Cell type(s) where they were shown to negatively regulate TNTs	References
Rac1	Actin dynamics, Rho GTPase	RAW/LR5, NIH3T3, MDA-MB-231, HUVEC		[111,82]
Cdc42	Actin dynamics, Rho GTPase	RAW/LR5, SH-SY5Y, H295R, MDA-MB-231, HUVEC	CAD	[112,111,119,48]
ROCK	Actin dynamics, Rho GTPase effector	SH-SY5Y	Primary Astrocytes, Primary microglia, A549, human TM	[17,48,55,66,110]
Arp2/3 complex	Branched actin nucleation	RAW/LR5, SH-SY5Y, human TM, U87, MDA-MB-231, HUVEC	CAD, U87	[111,55,54,48,66]
WASP	Actin dynamics, Arp2/3 activator downstream of Cdc42	RAW/LR5, SH-SY5Y		[111,48]
WAVE2	Actin dynamics, Arp2/3 activator downstream of Rac1	RAW/LR5		[111]
βCamKII	Actin bundling and stabilization	CAD, primary neurons		[120]
Cofilin	Actin severing an turnover	U2OS, HeLa, RPE1, SH-SY5Y, MCF-7		[121,122]
PAK1	Actin dynamics, Rho GTPase-effector	SH-SY5Y		[70,123]
VASP	Actin filament elongation		CAD	[112]
Myosin II	Cortical actin tension regulation	SH-SY5Y	B-cell, U-87, NRK,	[17,48,66,124,125]
M-Sec (TNFAIP2)	Exocyst complex recruitment and organization	M-cells, HeLa, U2OS, U87, primary AML cells, podocytes		[46,77,114,126–128]
Miro-1	Mitochondria fission, fusion, and trafficking	Rat primary astrocytes, PC12, LA-4, MSC, 3T3, NHBE, SMCs, neural stem cells		[19,21,129,130,38,44]
Miro-2	Mitochondria fission, fusion, and trafficking	Cardiomyocytes		[131]
MICAL2PV	Actin depolymerization	A549		[40]
Rhes	Membrane and vesicle dynamics	STHdh, mouse primary neurons		[73,75]
Eps8	Actin bundling and capping	CAD		[54,112]
IRSp53	Membrane-curvature-sensing scaffolding protein, and actin regulation	COS-7, A20 mouse B, CAD		[54,132]
Moesin	Membrane-actin crosslinking		Osteoclast precursors	[133]
Myo1D	Vesicle trafficking	Mouse primary pericytes, mouse primary astrocytes		[65]
Myo10	Filopodia formation and cargo transport	CAD, RAW 264.7, CEM-T4, TM, osteoclast precursors		[117,118,125,134]
Rab8a/Rab11a	Vesicle trafficking	SCs, MDCK, A549		[79,116,135]
Rab35 and associated proteins (ACAP2, MICAL-L1, ARF6, EHD1)	Vesicle trafficking	CAD		[49]
RalGPS2 and interacting proteins (Akt, PDK1, LST1, RalA)	Actin dynamics	Primary bladder cancer cells		[94,136,137]
Cell adhesion molecules (ICAM1)	Intercellular adhesion	T-cells		[41,138]
Transcription factor p53	Stress response regulation	Rat primary astrocytes, C6 glioma cells		[43]
MAPK pathway downstream of EGFR	Stress response regulation	A549, ovarian cancer cells, colorectal cancer cells		[92,110,139]
OXER1 receptor	Stress response regulation	H295R		[119]
HGF/c-Met/β1-integrin signaling pathway	Intercellular adhesion	A549		[110]
Tetraspanins (CD9, CD81)	Intercellular adhesion and membrane fusion	SH-SY5Y cells		[61]
N-cadherin	Intercellular adhesion	HEK293, C2C12		[48,140]
Connexin**-**43	GAP-junction formation	Astrocytes, neurons		[33]
FAK	Actin dynamics, cell-matrix adhesion	SCC-derived cells, primary astrocytes		[59,66]
Syncytin-1/2, MFSD2A recceptor	Membrane fusion	HEK293, MDA-MB-231, U2OS, A549, MCF5		[62]

Actin is the most abundantly expressed protein in mammalian cells and participates in a wide variety of cellular processes. The actin regulators mentioned above have pleiotropic functions, complicating efforts to identify their precise roles in TNT formation. Advanced tools, such as FRET- or split-GFP-based live reporters, are needed to directly visualize TNT fusion and formation dynamics, enabling high-resolution spatiotemporal correlation with protein and lipid activity. While actin dynamics provide the structural framework, TNT biogenesis also depends on the composition and organization of the plasma membrane, which influences membrane curvature, stability, and the recruitment of regulatory complexes. In the next section, we examine how membrane lipids and associated proteins contribute to TNT formation and function.

## Plasma membrane composition and TNT formation

The plasma membrane is a dynamic structure composed of phospholipids, glycolipids, cholesterol, and proteins, which collectively regulate fluidity, signaling, and membrane curvature. Several membrane-associated proteins have been linked to TNT formation (Table 2).

### Membrane scaffolding proteins

IRSp53, an I-BAR domain protein, is a membrane curvature sensor and adaptor that links Rho GTPases (e.g., Rac1, Cdc42) to actin regulators (e.g., N-WASP, WAVE2, Arp2/3) [141,142]. It enriches and stabilizes regions of negative membrane curvature and promotes protrusion formation. IRSp53 localizes to curved regions and facilitates both filopodia and TNT formation by recruiting actin polymerization machinery [54,142]. Supporting the present model, substrate-induced membrane nanodeformations can promote local actin polymerization via IRSp53-Cdc42 interactions [143].

M-Sec (TNFAIP2) is a central factor in TNT biogenesis. This protein localizes to the plasma membrane via its polybasic N-terminus binding o phosphoinositides (e.g., PI(4,5)P_2_), where it interacts with RalA and the exocyst complex, orchestrating actin-based membrane remodeling to drive TNT formation. Leukocyte-specific transcript 1 (LST1), a transmembrane scaffolding protein of the MHC class III family, acts as a scaffold by recruiting RalA, filamin, M-Sec, NMII, and myoferlin. Together, they assemble the multiprotein complex essential for TNT formation by actin and membrane remodeling [46,137,144–146].

Tetraspanins CD9 and CD81, known for their roles in membrane microdomain organization and fusion processes [147], have been found enriched in TNTs in U2OS cells and were, respectively, linked to TNT stability and functionality in SH-SY5Y cells [61].

### Cell adhesion proteins

The cell–cell adhesion protein N-Cadherin has been implicated in TNT stability or function in various cell types, including CAD, SH-SY5Y, HeLa, urothelial, U2OS, and HEK cells [10,48,61,140,148–150]. In SH-SY5Y cells, N-Cadherin binding partners α-Catenin and p120-Catenin have also been shown to promote TNT-mediated transfer [48].

Similarly, ICAM1 has been implicated in TNT-mediated transfer [51].

Cx43 down-regulation has been shown to reduce TNT formation in human trabecular meshwork and breast cancer cells [151,152]. Furthermore, Cx43 was demonstrated by down-regulation and overexpression to mediate TNT formation and mitochondrial transfer from human iPSCs and mouse epithelial cells [153], while up-regulation of functional Cx43 in murine primary astrocytes increased mitochondria transfer towards mouse primary neurons [33].

In A546 lung carcinoma cells, the hepatocyte growth factor (HGF), its receptor c-Met, as well as β1-integrin and paxillin, were shown to promote TNT-mediated transfer of lipid vesicles and mitochondria [110]. This pathway was shown to trigger downstream activation of MAPK and PI3K pathways and the Arp2/3 complex.

### Fusogens

Recently, the first *bona fide* fusogens, defined as both sufficient and necessary to drive fusion, have been implicated in the formation of functional TNTs (139): Syncytin-1 and -2, classically known for mediating cytotrophoblast fusion during placental development, were shown to be necessary for the TNT-mediated transfer of endosomal components as well as Cas9 proteins. This was found true for a range of cancer cell lines, such as HEK293T, MDA-MB-231, U2OS, A549, and MCF5. Similarly, knockdown of syncytin-2 receptor MFSD2A reduced Cas9 transfer in both HEK293T and MDA-MB-231 cells. It remains unclear whether TNT fusion across model systems is mediated by other known fusogens co-opted from other pathways or by proteins uniquely adapted to this process.

### Lipids

Although the specific lipid composition of TNTs remains poorly defined, several studies have begun to elucidate relevant features. Early work proposed that the lipid makeup of TNT membranes is likely important because of their unusual membrane architecture and mechanical requirements [154]. In a cancer-cell model, TNTs formed by urothelial tumor cells, but not by normal cells, were shown to be enriched in cholesterol/sphingomyelin-rich lipid-raft domains and display distinct elastic properties [155]. Consistent with that observation, in HEK293 cells, perturbation of cholesterol contents affected TNT mechanical stability [140]. Moreover, lipid droplets have been reported to be transported through TNTs in HMEC-1 endothelial cells and human astrocytes. In these models, elevated arachidonic acid levels, which promote lipid droplet biogenesis, correlated with increased TNT formation, underscoring the involvement of lipid-related organelles in TNT biology [87,88].

Recent studies have expanded this view, suggesting that specific membrane lipids not only influence TNT stability but also actively regulate membrane curvature and protein recruitment. The transmembrane proteins CD9 and CD81, enriched in U2OS TNTs and shown to regulate their functionality in SH-SY5Y cells, are established components of sphingolipid- and cholesterol-rich membrane microdomains [61]. Activation of the transmembrane protein CD13 has been shown to regulate phosphoinositide signal transduction during TNT biogenesis, involving FAK/Src signaling, small GTPases, and PI(4,5)P2 [156]. Additionally, the PI3K-Akt pathway has been implicated in TNT-mediated transfer, indirectly suggesting a role for PI(3,4,5)P_3_ [110].

Together, these data point to cholesterol-sphingomyelin-rich domains, curvature-sensing lipids, and mechanoelastic properties as key contributors to TNT formation and stability. However, systematic, quantitative lipidomic analyses of TNTs remain a major need in the field.

Overall, it emerges that actin dynamics and membrane composition together provide the physical and biochemical framework for TNT biogenesis. However, TNT formation is not merely a constitutive process. Rather, cells can markedly increase TNT formation in response to environmental cues. In the following section, we review how various cellular stresses can promote TNT formation by activating signaling pathways converging on the cytoskeletal and membrane remodeling machinery.

## TNT up-regulation in response to cellular stresses

Cells can increase TNT formation under stress, likely through signaling pathways that modulate both the cytoskeleton and membrane dynamics. Oxidative stress, hypoxia, inflammation, and infection have all been shown to enhance TNT formation, often via NF-κB-dependent mechanisms [16,36,66,114,157–160]. A recent report also links the actin-depolymerizing factor cofilin to oxidative stress-induced TNT formation in MCF7 cells [122].

In astrocytes, p53 promotes TNT formation in concert with Akt, phosphoinositide 3-kinase (PI3K), and mTOR [43]. In other cell types, however, TNT formation occurs independently of p53 expression [161], suggesting cell-type-specific control. One downstream p53-mediated mechanism involves activation of caspase-3, leading to cleavage of the metastasis-associated protein S100A4. The resulting decrease in intracellular S100A4, along with the associated extracellular concentration gradient, provides directional guidance cues for TNT growth between neurons and astrocytes [162]. Of importance, while many studies report that various stress inductions increase TNTs, only a few correlate such stresses with an increase in their functionality [42,43,63,159].

The influence of cell-specific responsiveness to stress on TNT induction remains largely unknown. Along the same lines, some cell lines transfer more material through TNTs than others [62], implying that differences in protein and lipid composition may modulate TNT biogenesis, although the underlying molecular determinants remain largely undefined.

## Fundamental principles of membrane fusion

Membrane fusion is a universal process in which two lipid bilayers merge into one. It occurs in diverse contexts: vesicle trafficking, exocytosis, endocytosis, mitochondrial fusion, fertilization, self-fusion, as well as syncytium formation following cytotrophoblast, myoblast, or osteoclast fusion.

In TNTs, membrane fusion is extremely poorly investigated, mainly due to the lack of efficient tools available to detect this process. Yet, fusion is arguably the most defining step in TNT biogenesis, as it enables membrane and cytoplasmic continuity, allowing active cargo transfer and rapid exchange via passive diffusion of membrane and cytosolic proteins and lipids, unique to TNTs.

**Figure 1:**
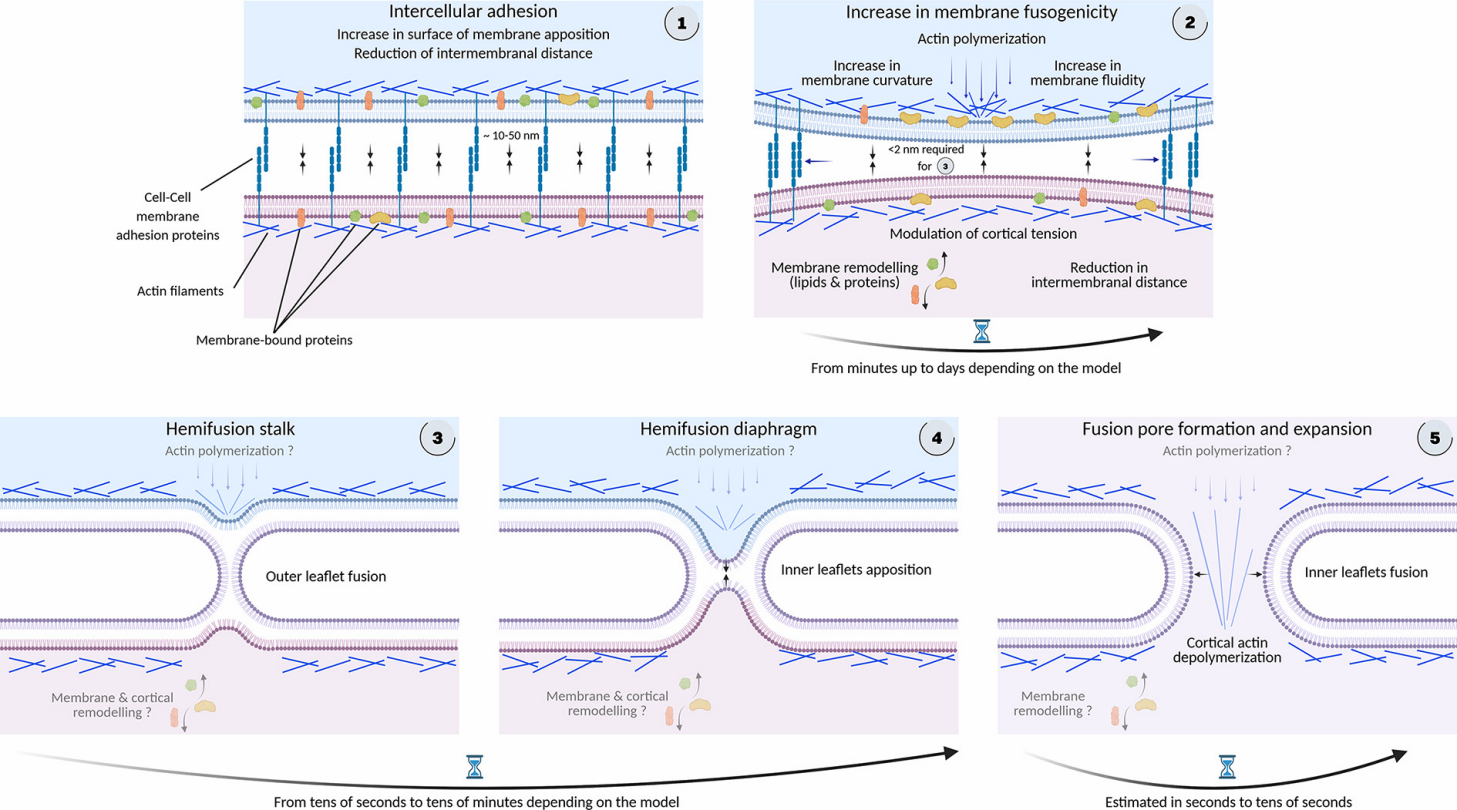
Common principles of cell-to-cell membrane fusion (**1**) Membrane fusion is facilitated by intercellular adhesion proteins, which reduce the distance between opposing membranes and increase the area of membrane apposition. (**2**) Pre-fusion membrane maturation increases membrane fusogenicity through lipid and protein remodeling, local clearance of adhesion molecules, and recruitment of protein complexes that drive actin cytoskeletal reorganization. Such cytoskeletal remodeling can be asymmetric, generating differential cortical tension and invasive actin digitations that exert orthogonal protrusive forces to promote fusion. Together, these changes tend to increase membrane fluidity and curvature and further reduce intermembrane distance. (**3**) Fusogens then catalyze the merger of the outer membrane leaflets, forming a hemifusion stalk. The precise contributions of actin and membrane remodeling beyond this step remain uncertain. (**4**) The distance between inner membrane leaflets narrows, leading to the formation of a hemifusion diaphragm. (**5**) Finally, fusogens mediate the fusion of the inner leaflets, leading to the formation of an unstable and often transient fusion pore. Cortical actin depolymerization has been shown to facilitate the expansion of the fusion pore. The temporal scale of these different steps is indicated. Refer to Table 3 for key factors regulating intercellular adhesion, actin remodeling, or membrane fusogenicity in well-characterized cell–cell membrane fusion models. Factors in red have been reported to promote TNT formation. Created with Biorender.com.

Before examining plasma membrane-to-plasma membrane fusion in TNTs, we first outline the fundamental principles of membrane fusion in mammalian cells (Figure 1 and Table 3).

**Table 3:** Actors with related functions across membrane fusion models

Membrane fusion model	Regulators of intercellular adhesion	Regulators of actin dynamics	Regulators of membrane fusogenicity	References
Sperm–egg fusion	IZUMO1, JUNO	**N-WASP, cofilin**, phosphoinositides	**CD9, cholesterol**, PS, phosphoinositides	[163–167]
Myeloid-derived multinucleated cells (macrophages, osteoclasts)	E-cadherin, CD47, MFR	**WASP, Arp2/3, eps8, IRSp53, rhoA**, moesin	DC-STAMP, **IRSp53**, OC-STAMP,	[133,168–174]
Myoblast fusion in mammals	**N-cadherin**	Tks5, **NMII, WASP, WAVE, Arp2/3, rac1, cdc42, eps8, IRSp53**, ezrin	Tks5, PS, CD9, CD81, annexin 1-5, KCTD10, myomaker, myomerger, **IRSp53**	[175–181]
Epithelial self-fusion	E-cadherin	**RhoA, ROCK, Cdc42, Rac1, Arp2/3, NMII**		[182,183]
*Caenorhabditis elegans* neuronal fusion		**WASP, Arp2/3**, spectraplakin	EFF-1, AFF-1, dynamin, ESCRT III, syntaxin-2	[184–190]
Placental cell fusion	E-cadherin, β-catenin, cadherin-11, **connexin 43**, ZO-1, syndecans, galectin 1/3	**WASP**, PKA, ezrin,	PS, PKA, **syncytin 1-2/MFSD2A**/ASCT1-2, annexin-5	[33,62,151,191–197]

In red are actors reported to regulate TNT formation.

Membrane fusion proceeds through a series of energetically demanding steps, well documented in several excellent reviews [198–201]. Each step is catalyzed by specific proteins called fusogens, which lower these energy barriers, making fusion energetically favorable. In addition, fusion is assisted in the early steps by adhesion molecules that bring membranes into close proximity and stabilize them. Actin cytoskeleton remodeling and motor proteins can generate local curvature and tension to destabilize bilayers, while certain lipids, particularly cone-shaped lipids, promote the negative curvature needed for fusion. Although fusogens are defined as proteins sufficient on their own to induce fusion of otherwise non-fusogenic membranes, in many systems, they likely act in concert with other proteins and lipids whose contributions remain poorly understood.

Fusion begins with membrane apposition (docking), in which membranes must be brought within distances below 2 nm of each other, therefore requiring the clearance of intercellular adhesion proteins. At this distance, closely opposed bilayers must overcome electrostatic repulsion between negatively charged phospholipid headgroups and the hydration force exerted by tightly bound water molecules (known as hydration shells). These hydration shells stabilize the membrane interface but resist compression.

The next stage is hemifusion, in which the outer leaflets merge while the inner leaflets remain distinct. This step requires membrane bending, often promoted by negative membrane curvature. It is characterized by lipid mixing without content mixing and is generally transient for cellular membranes, lasting from tens of seconds to tens of minutes. In C2C12 myoblast fusion, for example, hemifusion diaphragms have an average lifetime of 15 min. We can note that this duration is perfectly compatible with protein and membrane remodeling of the inner leaflet of the membrane, even if not demonstrated so far to our knowledge. In the extreme case of sea urchin fertilization, secretory cortical granules can remain in a hemifused state with the egg’s plasma membrane for weeks, suggesting that hemifusion intermediates can be exceptionally stable in adapted biological contexts.

Finally, the inner leaflets fuse to form a nascent fusion pore (1–2 nm wide), which can either close or expand within a matter of seconds. This final expansion step represents the most energy-demanding phase, overcoming membrane curvature stress and line tension at the pore edge. The expanded fusion pore can reach diameters of 10 to 30 nm. In syncytia, local actin depolymerization around the fusion site is thought to favor pore expansion, making full fusion energetically favorable.

Some of the best-studied examples of membrane fusion processes include synaptic vesicle fusion, enabled by SNARE fusogens [202]; mitochondrial fusion mediated by mitofusins 1 and 2 for the outer membrane and OPA1 for the inner membrane [203]; as well as viral fusion. Viral fusogens are numerous and fall into three structural classes (I–III) [204], each with distinct mechanisms. The influenza hemagglutinin and SARS-CoV-2 spike proteins, for example, are class I fusogens.

Here, we focus on plasma membrane-to-plasma membrane fusion in mammalian cells, as this is the type that must occur during TNT formation to achieve cytoplasmic continuity.

## Plasma membrane-to-plasma membrane fusion

### Sperm–egg fusion

In mammalian sperm–egg fusion, the IZUMO1 immunoglobulin-like protein on sperm is required for fusion by binding to the JUNO GPI-anchored receptor on the egg. Eggs lacking the tetraspanin CD9 can bind sperm but rarely fuse. CD9 is concentrated on microvilli, where curvature appears to increase fusogenicity. Cholesterol must be partially removed from the membrane to enable fusion, yet cholesterol-rich lipid rafts support fusion by clustering CD9 and JUNO; raft disruption impairs the process. During capacitation, a set of changes preparing the spermatozoid for fertilization, including membrane remodelling, phosphatidylserine (PS) is externalized from the inner to the outer leaflet of the sperm head membrane, promoting negative curvature. Finally, phosphoinositide PI(4,5)P_2_ modulates actin architecture and cortical remodeling in the egg by recruiting proteins such as N-WASP and cofilin, anchoring CD9 scaffolds to the membrane. PI(3,4,5)P_3_ is implicated in capacitation signaling prior to fusion [163–165,167].

### Virally induced syncytia

Viruses such as HIV and SARS-CoV-2 induce syncytia formation through their specific fusogens, in coordination with actin remodeling pathways dependent on Rac1 and RhoA. The events also involve cadherin-mediated cell–cell adhesion, which promotes the formation of multinucleated cells [205–208]. Interestingly, viral infection has also been shown to induce the formation of TNTs without necessarily triggering syncytium formation [8,81,157,209,210], while several lines of evidence suggest TNTs to be necessary for osteoclast fusion [211,212], indicating that these processes may rely on distinct underlying mechanisms.

### Macrophage and osteoclast multinucleation

As members of the mononuclear phagocyte system, macrophages and osteoclasts share most of their fusion machinery, including the dendritic cell-specific transmembrane protein (DC-STAMP), required for initiation and progression of fusion. E-cadherin up-regulation correlates with higher fusion efficiency in both cell types. Similarly, the macrophage fusion receptor (MFR) and its ligand CD47 have been implicated in the early stages of fusion by facilitating cell recognition and adhesion [169,170,173]. During macrophage syncytial formation, fusion requires Rho GTPases orchestrating actin rearrangements, with the involvement of WASP, ARP2/3, Eps8, and IRSp53 to form fusion-competent protrusions and stabilize the fusion pore. OC-STAMP appears essential for fusion in osteoclasts only and collaborates with DC-STAMP [168,171,172].

Of particular interest, TNT inhibition by latrunculin B treatment or M-Sec siRNA was shown to significantly reduce osteoclastogenesis [212]. More recent evidence suggests that TNTs actively promote fusion between osteoclast precursors. Cells forming thicker TNTs are more likely to fuse, and an increase in TNT formation following Moesin depletion correlates with enhanced fusion [211].

### Myoblast fusion

Myoblast fusion involves the fusogens myomaker and myomerger. Myomaker, which bears no resemblance to other known fusogens, appears necessary for hemifusion, while myomerger mediates pore formation. In mammals, N-Cadherin was shown to promote myoblast fusion, though many other adhesion proteins have been identified in *Drosophila*. PS exposure in the outer leaflet also acts as a fusion signal. NMII plays a key role by regulating cortical tension asymmetrically between fusing cells. Tetraspanins CD9 and CD81 promote fusion, as well as dynamin and annexins 1–5, possibly by facilitating curvature and pore expansion. A critical signaling cascade activating WASP/WAVE complexes to nucleate branched actin via Arp2/3, regulated by Rac1, Cdc42, Eps8, and IRSp53, has been identified [175–178,180]. Additionally, the ubiquitin ligase adaptor protein KCTD10 facilitates fusion by removing the Eps8-IRSp53 complex from the membrane in later stages [179]. Finally, the scaffolding protein Tks5 organizes actin regulators at fusion sites, forming invasive digitated structures with high membrane curvature at the tip.

Podosome-like structures exerting orthogonal protrusive forces at the fusion interface have been similarly observed in other fusion models. In non-fusing *Drosophila* S2R^+^ cells, overexpression of the *Caenorhabditis elegans* fusogenic protein EFF-1 was sufficient to trigger the formation of multinucleated cells [213]. This effect increased by nearly sevenfold when EFF-1 was coexpressed with the exogenous immunoglobulin domain-containing transmembrane protein Sns, which facilitates membrane merging by inducing the asymmetric assembly of invasive actin digitations through the recruitment of WASP and Arp2/3.

### Epithelial self-fusion

During development, some epithelial tubes may fuse to remodel tissues or close gaps. In MDCK cells plated on microfabricated pillar arrays, spread around micropillars and fuse with themselves. This fusion process involves serial activation of RhoA, ROCK, Cdc42, Rac1, Arp2/3, E-cadherin, and NMII-mediated contractility [182,183].

### *Caenorhabditis elegans* neuronal fusion

Neuronal fusion in *C. elegans* has been successfully characterized in the last decades, with the identification of many actors, including two fusogens: EFF- and AFF-1 [184,186,187]. Furthermore, actin regulators such as WASP and Arp2/3 have been shown to promote fusion [190], which appears to be dependent on the actin-binding protein spectraplakin, which also binds EEF-1 [189]. Dynamin, syntaxin-2, and ESCRT III have been involved in fusion by similarly regulating fusogen recruitment [185,188].

### Placental fusion

Trophoblast fusion is driven by the fusogens syncytin-1/-2 and their receptors ASCT1/2/MFSD2A, with knockdown directly reducing multinucleation [193,194]. Stable cell–cell contacts mediated by E-cadherin [214], β-catenin [215], cadherin-11 [216], connexin-43 [197,217], ZO-1 [218], syndecans [219], and galectins 1/3 [220,221] organize membranes for fusion. Annexin-A5 stabilizes fusogenic membrane domains [195], while PS exposure, mediated by TMEM16F [222], lowers the energy barrier for membrane merger. Actin and cortical remodeling are coordinated by N-WASP, Ezrin, and PKA signaling [190,215], promoting syncytin expression and fusion competence.

Of particular interest, Vargas et al. showed that syncytin-2-dependent fusion of human cytotrophoblasts correlates with the formation of microvilli enriched in early endosomes and pro-fusogenic proteins and that inhibition of microvillus formation through Ezrin inhibition blocks fusion [192].

## From general fusion to TNT-specific mechanisms

The examples above illustrate the diverse strategies cells use to overcome the energetic and structural barriers to membrane fusion, from specialized fusogens to lipid rearrangements and actin-driven remodeling. TNTs are unusual among plasma membrane-to-plasma membrane fusion events in that they often form between different cell types, in non-adherent conditions, and over long distances, all of which could influence the fusion machinery engaged. Moreover, TNT fusion does not result in syncytia formation. Importantly, direct evidence of TNT fusion remains extremely scarce [10], with most conclusions drawn indirectly from observations of cargo transfer, leaving fundamental mechanistic questions unresolved. Consequently, rather than being identified through fusion assays, candidate regulators in TNTs have largely been inferred from their roles in TNT formation and in other fusion systems.

In the following section, we examine current evidence for membrane fusion in TNTs, highlight methodological challenges in detecting and quantifying it, and discuss candidate molecules and models based on analogies with better-characterized fusion systems (Table 3).

### Candidate regulators of fusion in TNTs

Although numerous factors have been proposed to influence TNT formation, their implication in fusion has been difficult to assess, primarily due to the lack of suitable detection tools. TNT formation, however, has been associated with the fusion of osteoclast precursors [211,212]. In well-characterized fusion systems, the fusion event is relatively easy to identify, often involving complete membrane merging that produces multinucleated cells visible by confocal microscopy. In the case of MDCK self-fusion, micropillar arrays can even predict fusion sites, which are likewise observable with confocal imaging [182,183]. By contrast, TNTs still lack equivalent methodologies that would allow precise determination of when and where fusion occurs and, consequently, how it correlates with local physical constraints, lipid or protein distribution, and protein activity.

Common players and principles overlap between fusion processes, and some players identified in TNT-mediated transfer are known fusion facilitators. Cadherins, for instance, promote TNT-mediated transfer and/or TNT stability and appear to promote fusion in various models by promoting adhesion while maintaining membrane distances that must ultimately be overcome for fusion. Even in virally induced syncytial formation, cadherins regulate membrane apposition surfaces and fusion efficiency [182,208,183]. In SH-SY5Y, increased cell density promoted by N-cadherin enhances TNT-mediated transfer cells, where N-cadherin also stabilizes the iTNT bundle [48].

The highly curved membranes of TNTs resemble the actin-based digitations described in myoblast fusion [180,213], the ezrin-dependent microvilli necessary for cytotrophoblast fusion [192], and the tetraspanin-rich microvilli where sperm–egg fusion occurs [223]. Actin remodeling has been shown to regulate fusion pore size and kinetics in all previously described fusion models. Branched actin nucleator complex Arp2/3 and its upstream activators Rac1 and Cdc42 play a pivotal role in generating fusion-competent membrane protrusions in myoblasts, macrophages, sperm–egg fusion, osteoclast fusion, and virally induced syncytia. NMII has been shown to regulate fusion pore opening and to generate asymmetric cortical tension during fusion, similar to its role in TNT stability. WASP is necessary for fusion in macrophages and osteoclasts and for TNT formation in macrophages. Similarly, tetraspanins CD9 and CD81 promote sperm–egg and myoblast fusion and also regulate TNT formation and cargo transfer.

Furthermore, observations in HeLa cells suggest that PI(4,5)P_2_ and/or PI(3,4,5)P_3_ are associated with M-Sec-induced TNT formation [127]. Specifically, PI(4,5)P_2_ was localized along M-Sec-positive TNTs, whereas PI(3,4,5)P_3_ predominantly accumulated within plasma membrane ruffles at the cell periphery.

The proteins and lipids highlighted above participate in a wide array of signaling pathways and cellular processes, making it challenging to disentangle their specific contributions to TNT fusion. Despite their recurrent implication in TNT biogenesis and membrane fusion-related mechanisms, as previously stated, determining whether they specifically influence membrane fusion requires the development of refined tools and experimental protocols.

In this context, the recent identification of the *bona fide* fusogens syncytin-1 and syncytin-2 as mediators of TNT-dependent Cas9 transfer between multiple cancer cell types represents a major advance for the field [62].

Ultimately, pinpointing the molecular trigger of TNT membrane fusion remains one of the most pressing and technically challenging frontiers in the field, and progress will hinge on real-time visualization methods capable of capturing this elusive event in its native cellular context.

## Key questions

Several open questions remain regarding fusion mechanisms in TNTs, particularly when compared with better-characterized fusion models:

- Does fusion occur at the TNT tip or along its lateral surface?
- If tip fusion establishes cytoplasmic continuity between cells, what prevents the formation of a permanent syncytium?
- In the absence of mechanical stress, would a TNT remain open indefinitely, or is closure an intrinsic, energetically favorable property?

**Figure 2:**
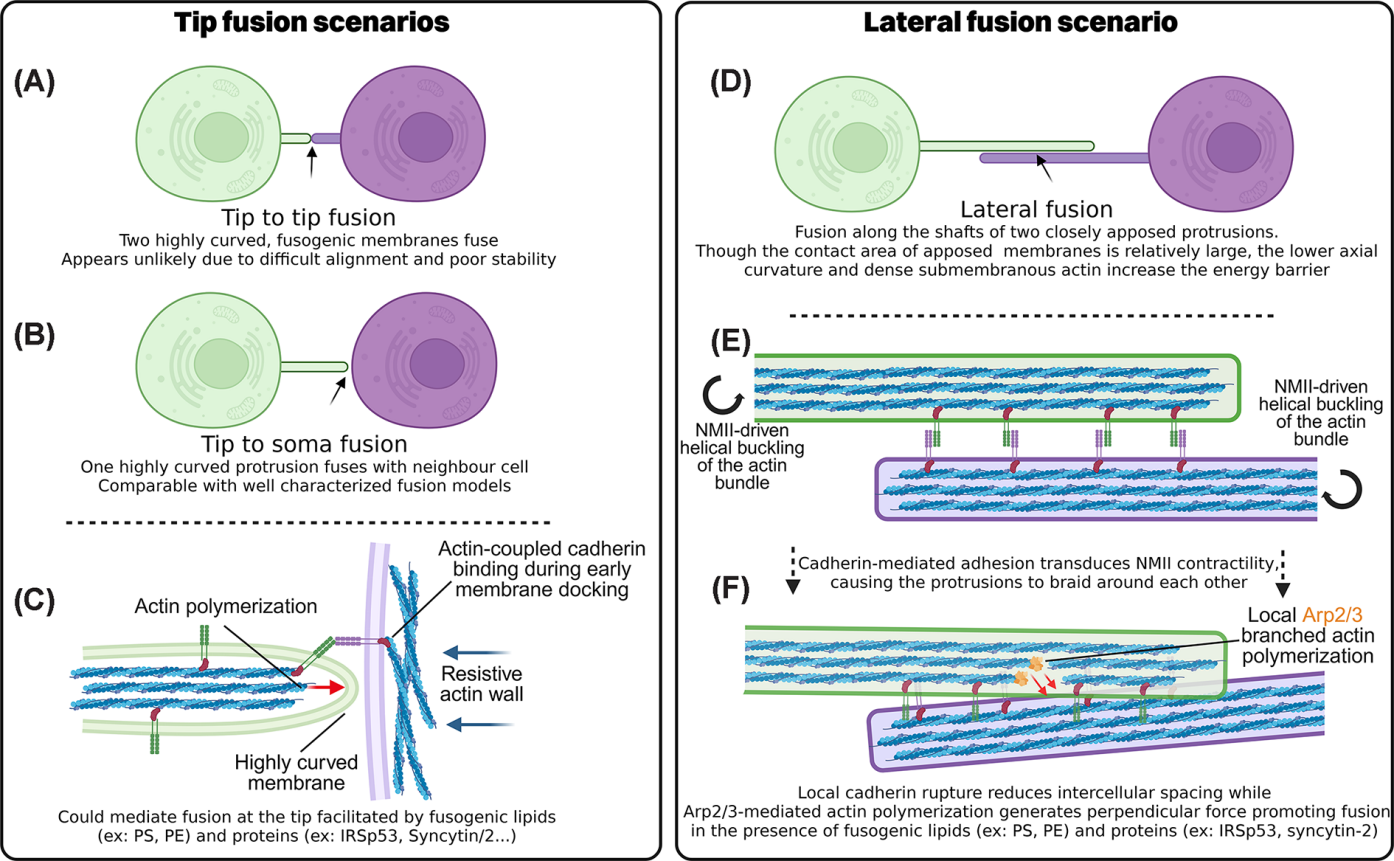
Hypothesized mechanisms of tip-mediated and lateral fusion during TNT formation (**A,B**) Schematics illustrating two potential modes of tip-to-target fusion during TNT formation. In both cases, fusion is proposed to occur at highly curved filopodial tip. (**C**) Zoomed view highlighting the geometry of tip to soma fusion, illustrating how membrane curvature, actin organization, and perpendicular forces generated by actin polymerization may contribute to fusion. Refer to Figure 1 for more mechanistic detail. (**D**) Schematic of a hypothetical lateral fusion pathway between closely apposed iTNTs. Despite a larger contact area, lateral fusion is considered less likely due to lower axial curvature and dense submembranous actin. (**E**) However, lateral fusion could be promoted by NMII-driven contractility transmitted through cadherin-based adhesions linking adjacent iTNTs, causing the protrusions to braid around one another and increasing local mechanical stress at contact sites. (**F**) Local rupture of cadherin adhesions reduces intermembrane distance, while Arp2/3-mediated branched actin polymerization within one iTNT generates a protrusive force oriented at ∼70° relative to the shaft axis, producing a perpendicular push that may promote membrane apposition and fusion. Figure created with Biorender.com.

In myoblast fusion, asymmetric cortical tension and orthogonal actin polymerization act together to drive membrane merging. A similar mechanism could be hypothesized at the tip of TNTs. However, lateral fusion between two juxtaposed tubes remains an equally plausible scenario (Figure 2). The bundled architecture of iTNTs offers extensive lateral membrane-membrane contact, and their high curvature may itself promote fusogenicity. In this context, NMIIA-driven contractility, by introducing torsional forces and promoting membrane braiding, may reduce the intermembrane spacing and facilitate fusion. Local Arp2/3-mediated F-actin nucleation at a ∼70° angle could provide the oblique protrusive force necessary to bring the two iTNT membranes into close proximity. Indeed, Arp2/3 and its upstream regulators have been implicated in promoting TNT formation and/or TNT-mediated cargo transfer [48,55,110,111]. Furthermore, live imaging in microglial cells revealed functional transfer through a branched TNT [16], suggesting that Arp2/3-driven branched actin nucleation may also occur locally within TNT shafts, where it could contribute to lateral fusion.

From a biophysical standpoint, fusion pore expansion, a key step in both vesicle and cell–cell fusion, is energetically prohibitive below diameters of ∼30 nm and requires local actin depolymerization to increase membrane fluidity. Intriguingly, TNTs, which may be several hundred nanometers wide, rarely lead to multinucleated syncytia, suggesting additional regulatory barriers. The actin bundles within TNTs, stabilized by complexes such as Eps8-IRSp53 and possibly the membrane-cytoskeleton linkers such as ERM proteins (Ezrin, Radixin, and Moesin), may help maintain tube integrity and prevent complete fusion. This is reminiscent of filopodia, which, despite experiencing high line tension at their base, remain structurally stable due to their rigid actin cores. Supporting this notion, the recent observation that Moesin depletion enhances osteoclast precursor fusion suggests ERM-mediated actin-membrane linkage can restrict full cytoplasmic merging [211].

Another unresolved issue is the diffusion of cytosolic proteins within the densely packed TNT lumen. While diffusion barriers could, in some instances, impede free movement, live imaging has revealed rapid diffusion of large cytosolic proteins [3,4]. Yet these transfers most often cease spontaneously, suggesting instabilities in the TNT pore. Whether this necessitates dedicated fission machinery remains unclear. Actin retraction at one end of the TNT could locally reduce axial stiffness, generating uneven tension gradients along the curved shaft. Such conditions may favor necking and spontaneous fission, akin to the collapse of a soap film cylinder. In this context, the stability of the fusion pore may depend on F-actin integrity, which could act as a mechanical spacer preventing pore collapse. NMII-mediated contractility at the base of the shaft, by driving actin retrograde flow and local actin depletion, may therefore promote pore closure at the distal tip. However, optical tweezer experiments pulling membrane tubes from cells show that such tubes can remain mechanically stable under low tension. In fact, cortical actin depolymerization at the tube base tends to reduce membrane tension, thus enhancing tube stability.

## Conclusions

A growing number of proteins have been implicated in TNT-mediated intercellular transfer, many of which regulate actin dynamics, membrane curvature, cortical tension, and cell–cell adhesion. While *bona fide* fusogens such as syncytin-1 and syncytin-2 may be required for actual membrane merging, TNT formation and fusion are likely governed by many molecular and mechanical factors that both promote and restrain fusion, which can also be tissue specific. As in other membrane fusion processes, the coexistence of overlapping, partially redundant mechanisms may provide robustness and tight spatiotemporal control. However, directly linking the activity, localization, or regulation of these candidate proteins to membrane fusion events within TNTs remains a major challenge, largely due to the absence of tools capable of detecting fusion in living cells.

Addressing this gap will require the integration of advanced imaging approaches, genetically encoded biosensors, and targeted perturbations to resolve the sequence of events leading from TNT initiation to membrane fusion. In particular, the development of live assays, such as split-GFP- or FRET-based reporters that directly reveal cytosolic continuity following fusion, has the potential to transform the field by enabling functional discrimination between mere structural contacts and true open-ended connections.

From a biological perspective, the implications of TNT-mediated fusion are substantial. Even rare or transient fusion events could permit partial cytoplasmic equilibration between cells, buffering oxidative stress, reducing stochastic noise in gene expression, or shaping developmental and homeostatic gradients. Importantly, current *in vivo* evidence remains fragmentary, and the prevalence of TNTs in tissues is still unknown. Yet, even the exchange of a small fraction of cytosolic content, for example, on the order of a few percent, could have significant physiological consequences, conferring strong selective advantages. The likelihood that such events remain largely invisible to standard cell biological techniques highlights a critical limitation in our ability to capture dynamic, low-frequency intercellular processes in their native contexts.

## Perspectives

- TNTs as a distinct mode of intercellular communication: Tunneling nanotubes constitute a unique form of long-range cell–cell interaction, enabling the exchange of organelles, proteins, and signals in ways that may profoundly influence development, tissue homeostasis, immune responses, and disease progression.
- Membrane fusion remains the central unresolved mechanism: Although the molecular pathways driving TNT initiation and elongation are increasingly well characterized, the fundamental process of membrane merging during TNT biogenesis and maturation remains poorly understood. In particular, the lack of direct visualization of fusion events, whether occurring at nanotube tips or along the shaft, continues to limit mechanistic insight and fuels ongoing debate about TNT functionality.
- New tools will be essential to define TNT function *in vivo*: The development of live-cell fusion reporters and quantitative imaging strategies, such as split-GFP or FRET-based assays, will be crucial to directly detect TNT fusion, distinguish structural from functional continuity, and determine when and where TNTs contribute meaningfully to physiological and pathological processes.

## Open Access

Open access for this article was enabled by the participation of Institut Pasteur in an all-inclusive Read & Publish agreement with Portland Press and the Biochemical Society.

## Abbreviations

CAD: Cath.a-differentiated
DC-STAMP: dendritic cell-specific transmembrane protein
DNTs: dendritic nanotubes
HGF: hepatocyte growth factor
iTNTs: individual TNTs
LST1: leukocyte-specific transcript 1
MFR: macrophage fusion receptor
NMII: non-muscle myosin II
PS: phosphatidylserine
PI3K: phosphoinositide 3-kinase
TNTs: tunneling nanotubes
***Bona fide* fusogen**: A protein that is necessary and sufficient to catalyze membrane fusion by lowering energetic barriers to bilayer merger.
**Closed-ended TNTs**: Actin-based intercellular connections that terminate in apposed membranes connected by gap junctions, enabling electrical or small-molecule coupling within the size limit of gap junction pores (∼1.5 nm).
**Cone-shaped lipids**: Lipids (e.g., phosphatidylethanolamine) that promote negative membrane curvature and lower energetic barriers to fusion intermediates.
**DNTs**: Nanotube-like connections are described between neurons *in vivo*, implicated in calcium signaling and pathological protein spread, with unresolved membrane continuity.
**Fusion pore**: Nanoscale opening formed after hemifusion that allows content mixing; can either close transiently or expand to complete fusion.
**Hemifusion**: Intermediate fusion state in which outer membrane leaflets merge while inner leaflets and cytoplasmic contents remain distinct.
**I-BAR domain proteins**: Membrane curvature-sensing and -inducing proteins (e.g., IRSp53) that stabilize negatively curved membranes and link membrane shape to actin polymerization.
**iTNTs**: Thin membrane tubes (∼50–200 nm diameter) that can bundle together to form structures appearing as a single TNT by light microscopy.
**Lipid rafts**: Cholesterol- and sphingolipid-enriched membrane microdomains are implicated in clustering fusogenic proteins and regulating membrane mechanics.
**Membrane fusion**: Process by which two lipid bilayers merge into one, involving docking, hemifusion, fusion pore formation, and pore expansion.
**NMII**: Actin-based motor protein generating cortical tension and contractile forces.
**(Open-ended) TNTs**: Thin, submicrometer-wide actin-based membranous protrusions that connect distant cells and can establish direct cytoplasmic continuity through membrane fusion, enabling the transfer of vesicles, organelles, and passive diffusion of cytosolic components between cells.
**Phosphoinositides (PI(3,4,5)P_3_/PI(4,5)P_2_)**: Minor but highly regulatory membrane lipids that recruit actin regulators and scaffold proteins involved in TNT formation and fusion.
